# Comparison of efficacy and safety between oral and intravenous administration of tranexamic acid for primary total knee/hip replacement: a meta-analysis of randomized controlled trial

**DOI:** 10.1186/s13018-019-1528-8

**Published:** 2020-01-20

**Authors:** Wei Ye, Yafang Liu, Wei Feng Liu, Xiao Long Li, Yanqiang Fei, Xing Gao

**Affiliations:** 1 Department of Orthopedics Medicine, Wujin People’s Hospital, YongNing North Road No. 2, Changzhou, 213000 Jiangsu Province China; 2 Department of Respiratory Medicine, Wujin People’s Hospital, Changzhou, 213000 China

**Keywords:** Oral, Intravenous, Tranexamic acid, Total knee/hip arthroplasty

## Abstract

**Background:**

Tranexamic acid (TXA) has been demonstrated to reduce blood loss following primary total knee and hip arthroplasty. This study aimed to compare the efficacy and safety of oral and intravenous tranexamic acid for primary total knee and hip arthroplasty.

**Methods:**

The PubMed, Embase, and Cochrane Library databases were searched for relevant studies published before June 20, 2019. Studies clearly reporting a comparison of oral and intravenous TXA were selected, and total blood loss (TBL), the decline in hemoglobin (DHB), deep vein thrombosis (DVT), intramuscular venous thrombosis (IVT), the length of hospital stay, and the transfusion rate were evaluated. The weighted mean differences and relative risks were calculated using a fixed-effects or random-effects model.

**Results:**

Ten studies involving 1140 (oral 557; intravenous 583) patients were included in this meta-analysis. There was no significant difference in terms of total blood loss, the decline in hemoglobin, the length of hospital stay, the incidence of DVT or IVT, or the transfusion rate between the oral and intravenous groups, and five studies reported that oral TXA was associated with a lower cost.

**Conclusion:**

Our research suggests that compared with intravenous use of TXA, the oral approach has similar clinical outcomes and is less expensive for total joint replacement patients.

## Introduction

Total knee/hip arthroplasty (TKA/THA) is a reliable surgical procedure for patients suffering from moderate to severe degenerative knee or hip joint diseases and osteoarthritis pain, but perioperative bleeding is a major challenge for surgeons. Previous studies have reported that the estimated blood loss was between 800 and 1800 ml [1, 2]. To overcome this problem, various methods are available to reduce perioperative blood loss, such as blood transfusions, tourniquet use, iron supplements, and the administration of anti-fibrinolytic drugs [3–6]. As we all know, transfusions extend not only the patient’s rehabilitation time but also the length of hospital stay. They incur a considerable cost and are also associated with the risk of transfusion reactions, infectious diseases, and inhibition of the immune system [7–9]. Tranexamic acid (TXA) is a synthetic agent that exerts its anti-fibrinolytic effects by inhibiting plasminogen. TXA inhibits plasminogen activation by binding plasmin to fibrin, which leads to clot stabilization and reduces blood loss. TXA is a simple, cheap, and effective drug for reducing perioperative blood loss in total knee arthroplasty regardless of whether it is applied directly to the surgery site or through intravenous administration, which has been reported in previous publications [10–14]. However, there is still no consensus about which route is the best method application of TXA for total joint replacement patients. This study aimed to compare the efficacy and safety of oral and intravenous tranexamic acid for primary total knee and hip arthroplasty patients.

## Materials and methods

### Search strategy

PubMed, Cochrane Library, and Embase databases were searched independently by two investigators to retrieve relevant studies published before June 20, 2019. The search criteria “tranexamic acid,” “TXA,” “total knee/hip arthroplasty,” “TKA/THA,” “total joint replacement,” “TKR/THR,” “oral,” and “intravenous” were used in key words for search. All studies selected were reviewed independently by the authors and examined for broadening the potential studies through the “related articles” function. Thus, the reference lists of the included articles were also manually checked to find relevant studies that were not found during the database searches.

Inclusion criteria are the following: (1) adult patients with knee or hip joint degenerative disease and received primary TKA/THA; (2) includes TXA oral and intravenous application groups; (3) examination includes any one among total blood loss, decline of hemoglobin (DHB), DVT, IVT, length of hospital stay, and transfusion rate. Exclusion criteria are the following: (1) Joint replacement is not for joint degenerative disease such as trauma, tumors, or bilateral joint replacement; (2) Study only report oral or intravenous group or case report; (3) Animal or laboratory study.

### Data extraction

Each article’s variables and outcomes of interest and assessment of the methodological quality were reviewed independently by two readers. If there was a difference of opinion, the problems were resolved through discussion and consensus. The methodological quality of the trials was assessed through the Cochrane Handbook for Systematic Reviews of Interventions 5.1.

### Statistical analysis

The statistical analysis was performed using Review Manager 5.1 for Windows System (Cochrane Collaboration, Nordic Cochrane Centre, Copenhagen, Denmark). Categorical dichotomous variables were analyzed with relative risks (RRs), continuous variables were assessed with the weighted mean difference, and *P* < 0.05 was considered statically significant; the 95% confidence intervals (CIs) were reported. Heterogeneity was considered significant if the *P* value was less than 0.1. The value of *I*^2^ statistics was used to assess the degree of heterogeneity (*I*^2^ < 25%, no heterogeneity; *I*^2^ = 25–50%, moderate heterogeneity; *I*^2^ > 50%, large or extreme heterogeneity); if *I*^2^ > 50%, a fixed-effects model was used. The presence of publication bias was assessed by a visual inspection of a funnel plot and the Begg and Egger tests (with *P* < 0.05 considered statistically significant).

## Results

### Literature search

**Fig. 1:**
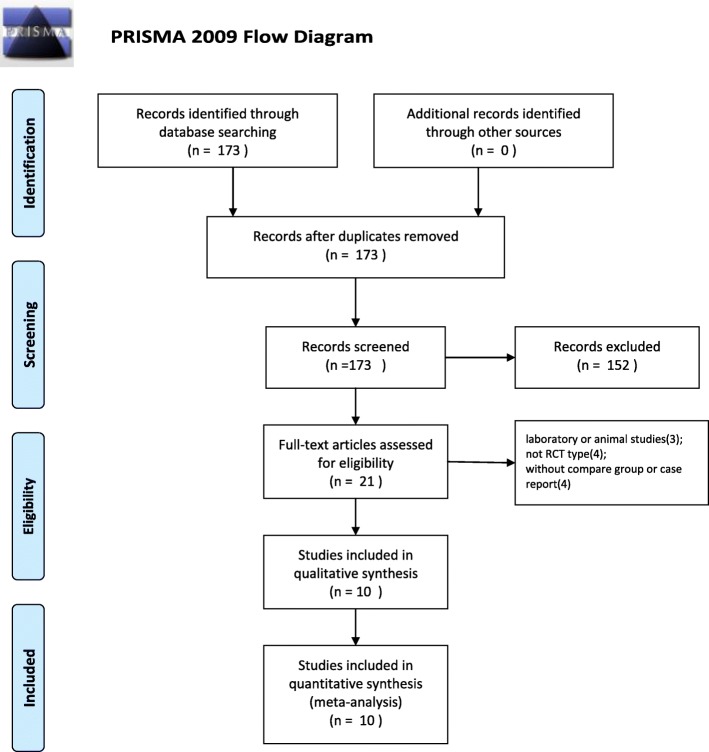
Search strategy flow diagram

**Fig. 2:**
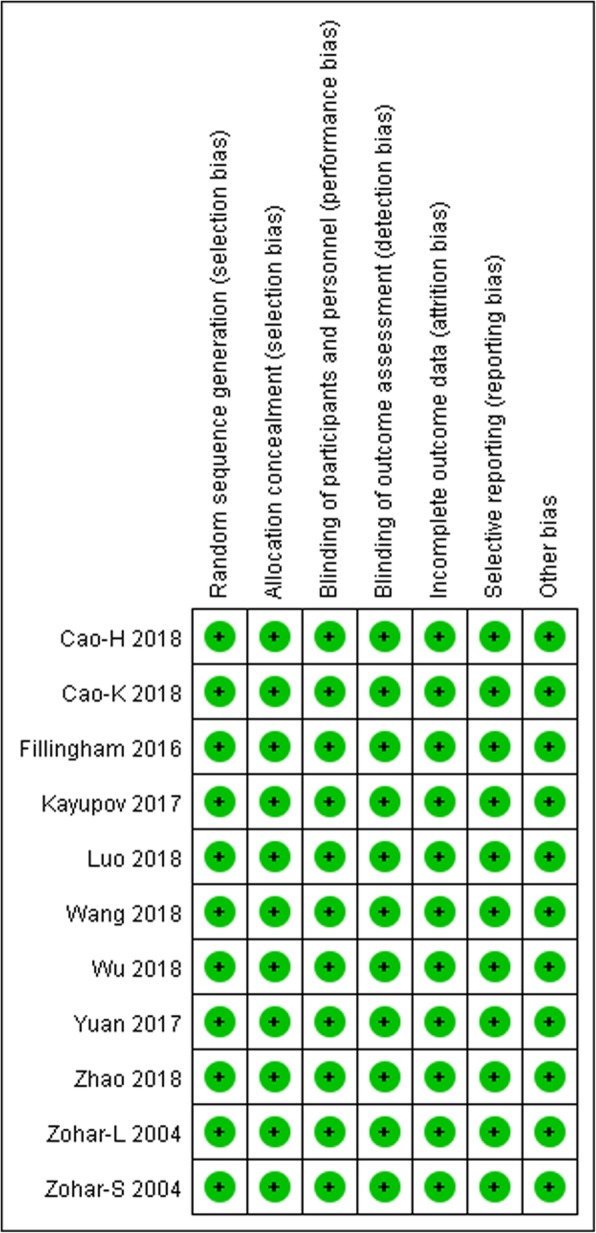
Summary of the methodological quality of the selected studies

The initial literature search retrieved 173 relevant articles (duplicates were discarded). After a careful screening of the titles, 152 articles were excluded for not investigating the topic of interest. After reviewing the abstracts, 11 articles were excluded (3 laboratory or animal studies, 4 not RCT type, 4 without compare group or case report). Therefore, leaving 10 studies matched the selection criteria and were suitable for meta-analysis (Fig. 1). A total of 1140 (oral 557; intravenous 583) patients were enrolled in the studies; the information of the included studies is summarized in Table 1 [15–24]. They are all RCT studies and the methodological bias of this study was low (Fig. 2).

**Table 1:** The information of included studies

Study	Country	Hip/knee	Oral	IV	TXA administration	TXA cost	Outcomes
Yuan 2017 [15]	China	K	140	140	O: 20 mg/kg*2 dose IV: 20 mg/kg*2 dose	O: $274.81 IV: $860.28	IVT, DVT, DHB, transfusion rate
Luo 2018 [16]	China	H	60	60	O: 2 g*1 dose IV: 20 mg/kg*1 dose	O: $68.11 IV: $472.44	TBL, DHB, transfusion rate
Wang 2018 [17]	China	K	60	60	O: 2 g*1 dose IV: 20 mg/kg*1 dose	O: $68.11 IV: $474.1	TBL, DHB, DVT, transfusion rate
Fillingham 2016 [18]	USA	K	34	37	O: 1950 mg*1 dose IV: 1 g*1 dose	NR	TBL, DHB, HS, transfusion rate
Wu 2018 [19]	China	H	50	50	O: 1 g*1 dose IV: 1 g*3 dose	O: $85.14 IV: $447	TBL, DHB, IVT, HS, transfusion rate
Cao-K 2018 [20]	China	K	59	59	O: 500 mg*4 dose IV: 20 mg/kg*1dose and 1 g*3 dose	NR	TBL, DVT, HS
Zhao 2018 [21]	China	H	40	40	O: 20 mg/kg*2 dose IV: 15 mg/kg*2 dose	O: $77.48 IV: $648.96	TBL, DHB, IVT, transfusion rate
Kayupov 2017 [22]	USA	H	40	43	O: 1950 mg*1 dose IV: 1 g*1 dose	NR	TBL, DHB, HS, transfusion rate
Cao-H 2018 [23]	China	H	54	54	O: 500 mg*4 dose IV: 20 mg/kg*1 dose	NR	IVT, DVT
Zohar-L 2004 [24]	Israel	K	20	20	O: 1 g*3 dose IV: 15 mg/kg*1 dose and 10 mg/kg*1 dose	NR	HS, transfusion rate
Zohar-S 2004 [24]	Israel	K	20	20	O: 1 g*3 dose IV: 15 mg/kg*1 dose and 10 mg/kg*1 dose	NR	HS, transfusion rate

*DVT* deep vein thrombosis, *IVT* intramuscular venous thrombosis, *DHB* decline of hemoglobin, *TBL* total blood loss, *HS* length of hospital stay

### Main analysis

**Fig. 3:**
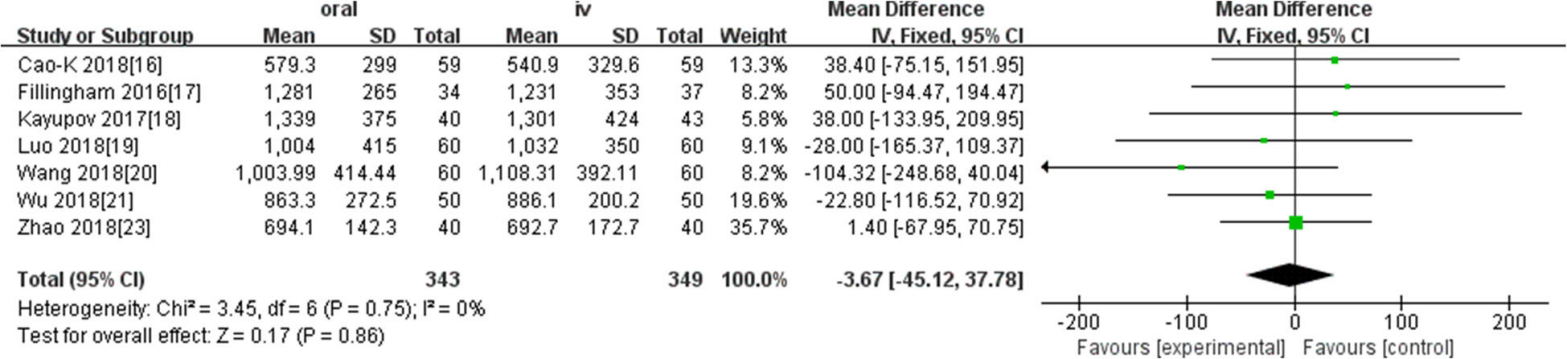
Forest plot showing the weighted mean difference in total blood loss between oral and IV groups

**Fig. 4:**
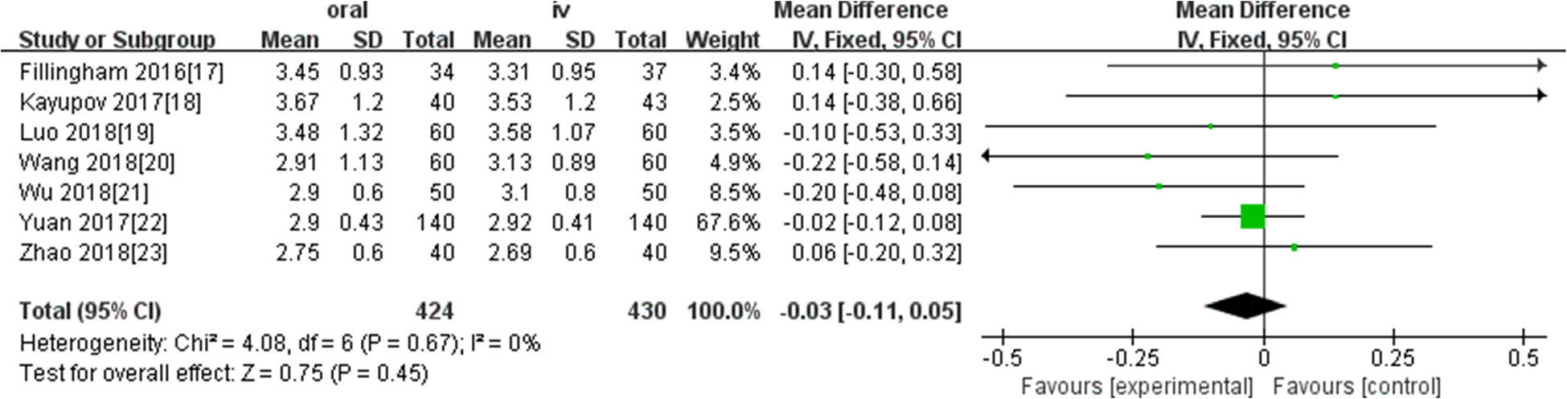
Forest plot showing the weighted mean difference in decline of hemoglobin (DHB) between oral and IV groups

**Fig. 5:**
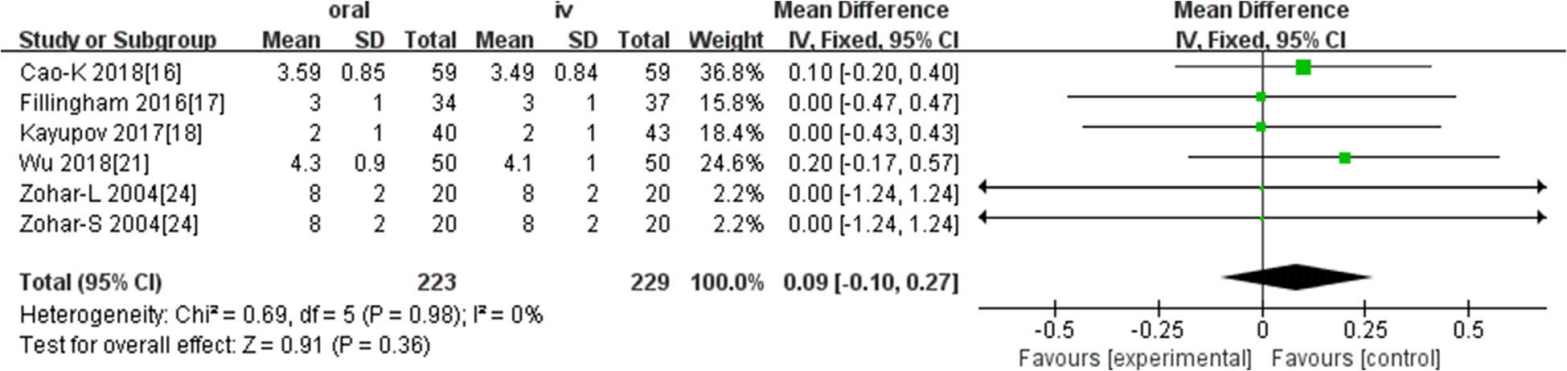
Forest plot showing the weighted mean difference in the length of hospital stay (HS) between oral and IV groups

**Fig. 6:**
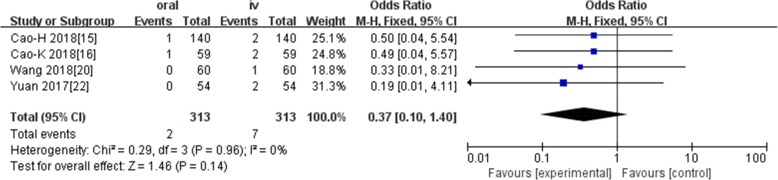
Forest plot showing the OR difference in DVT rate between oral and IV groups

**Fig. 7:**
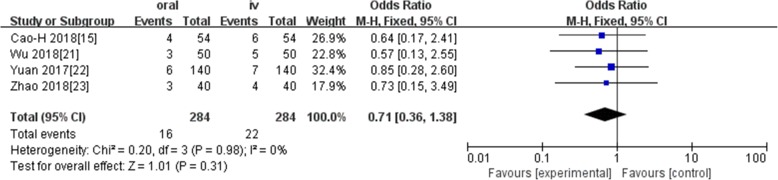
Forest plot showing the OR difference IVT rate between oral and IV groups

**Fig. 8:**
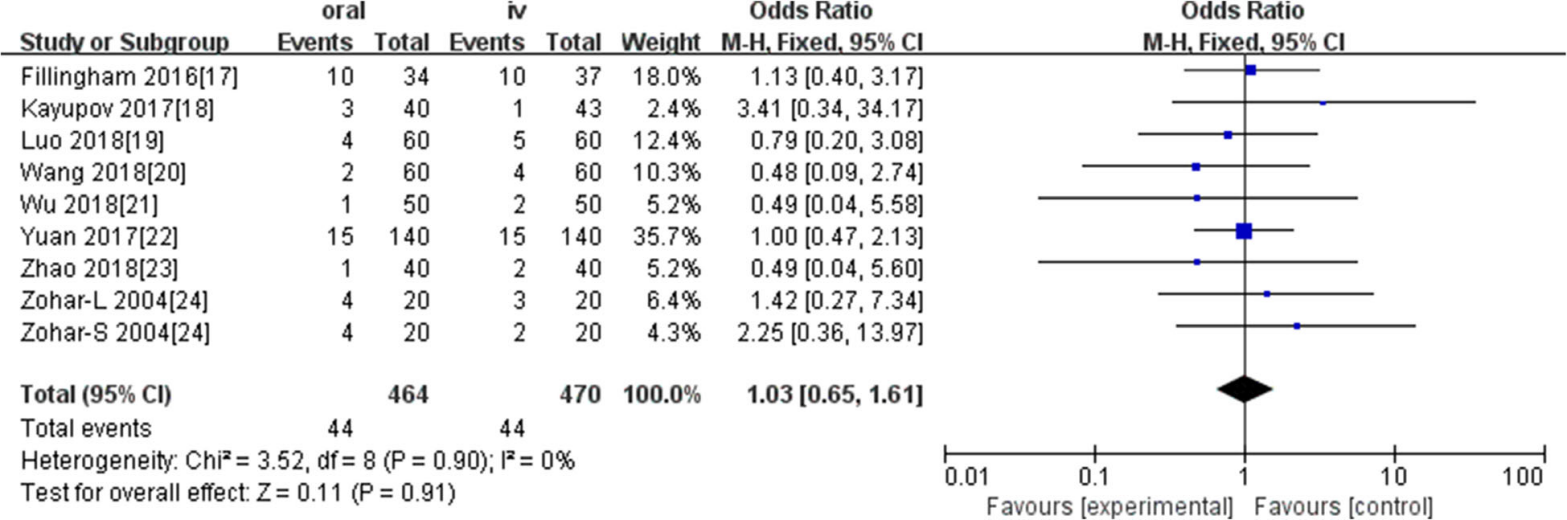
Forest plot showing the OR difference in transfusion rate between oral and IV groups

**Fig. 9:**
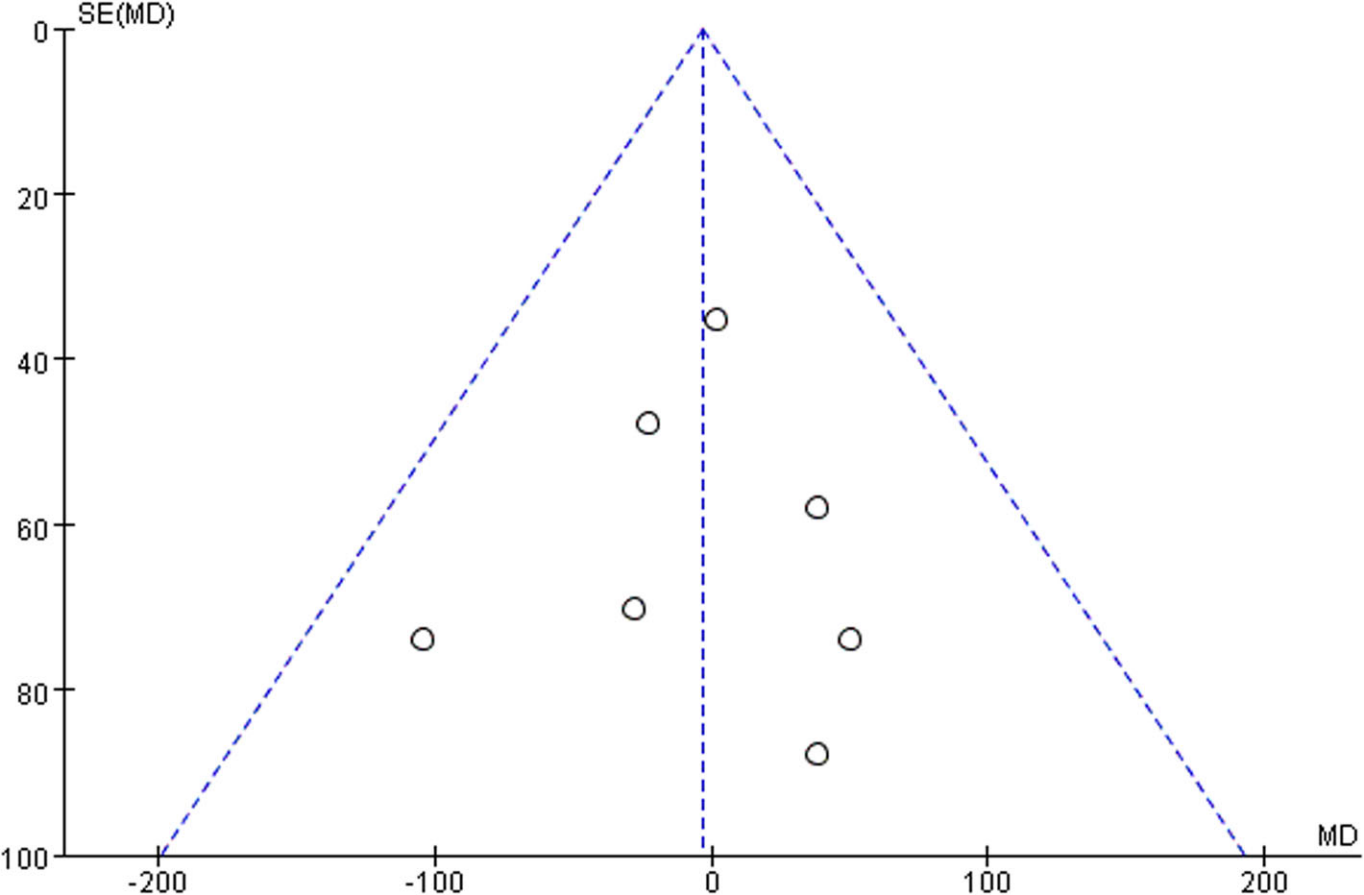
TBL funnel plot

**Fig. 10:**
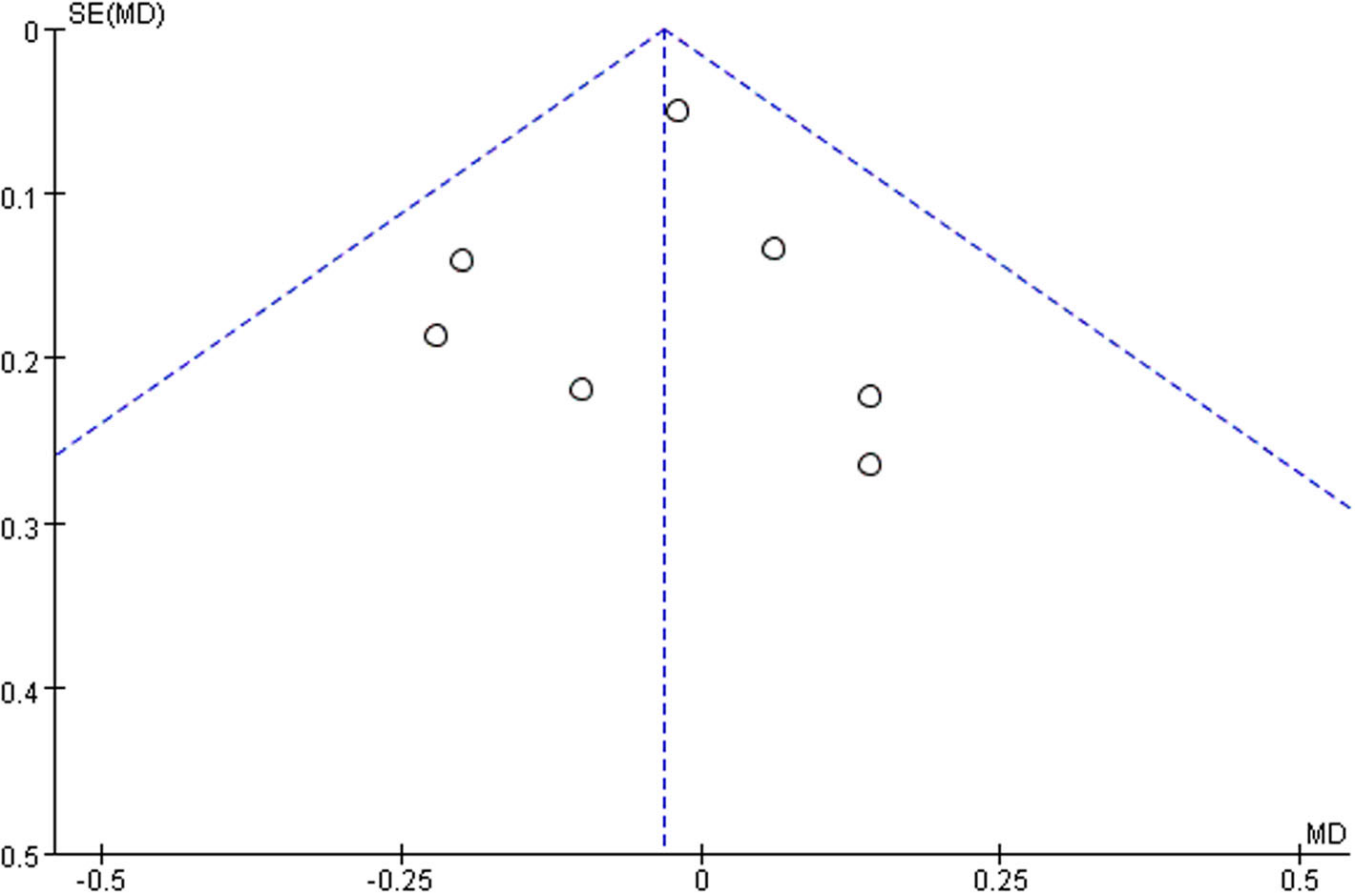
DHB funnel plot

**Fig. 11:**
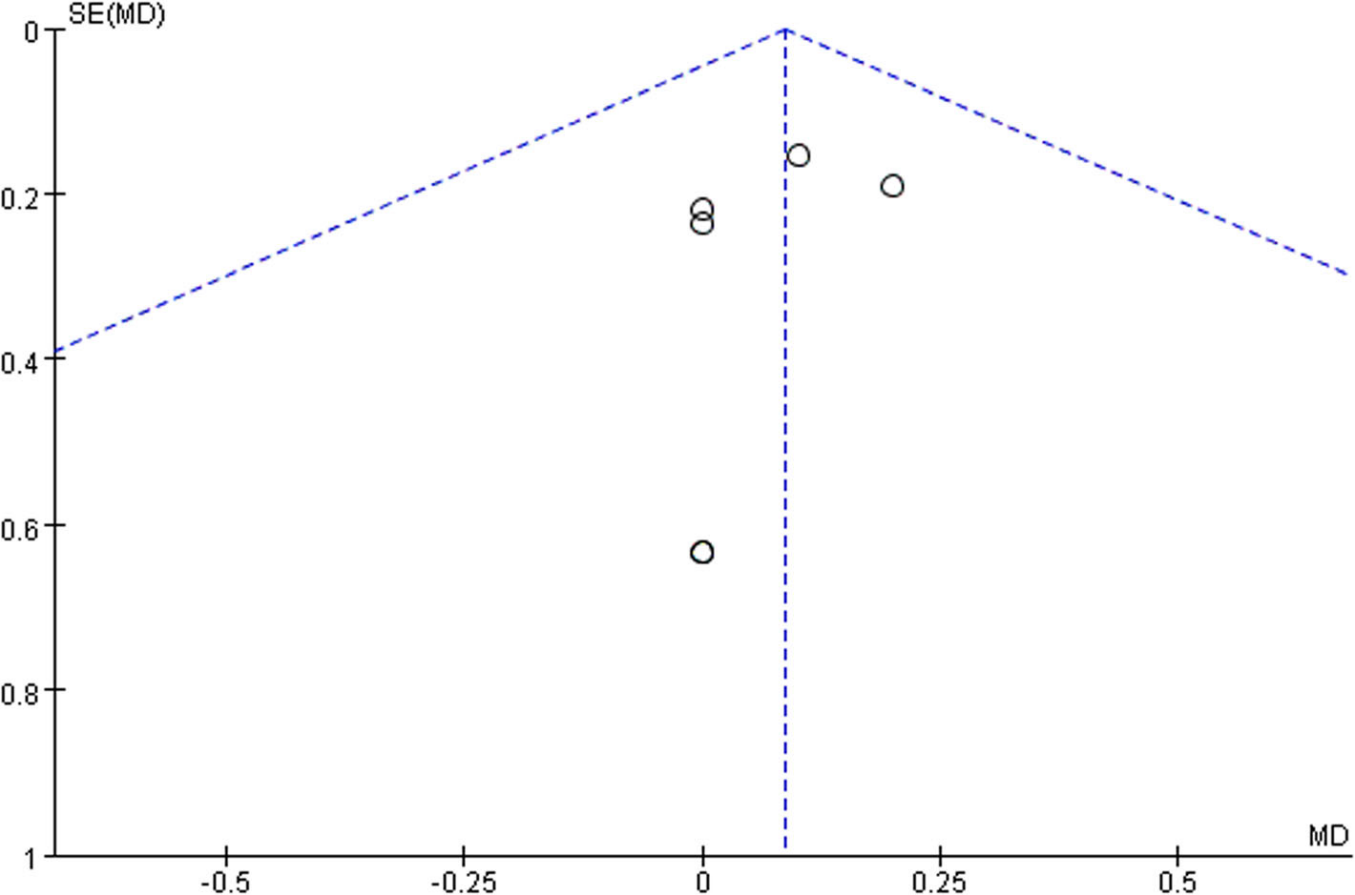
HS funnel plot

**Fig. 12:**
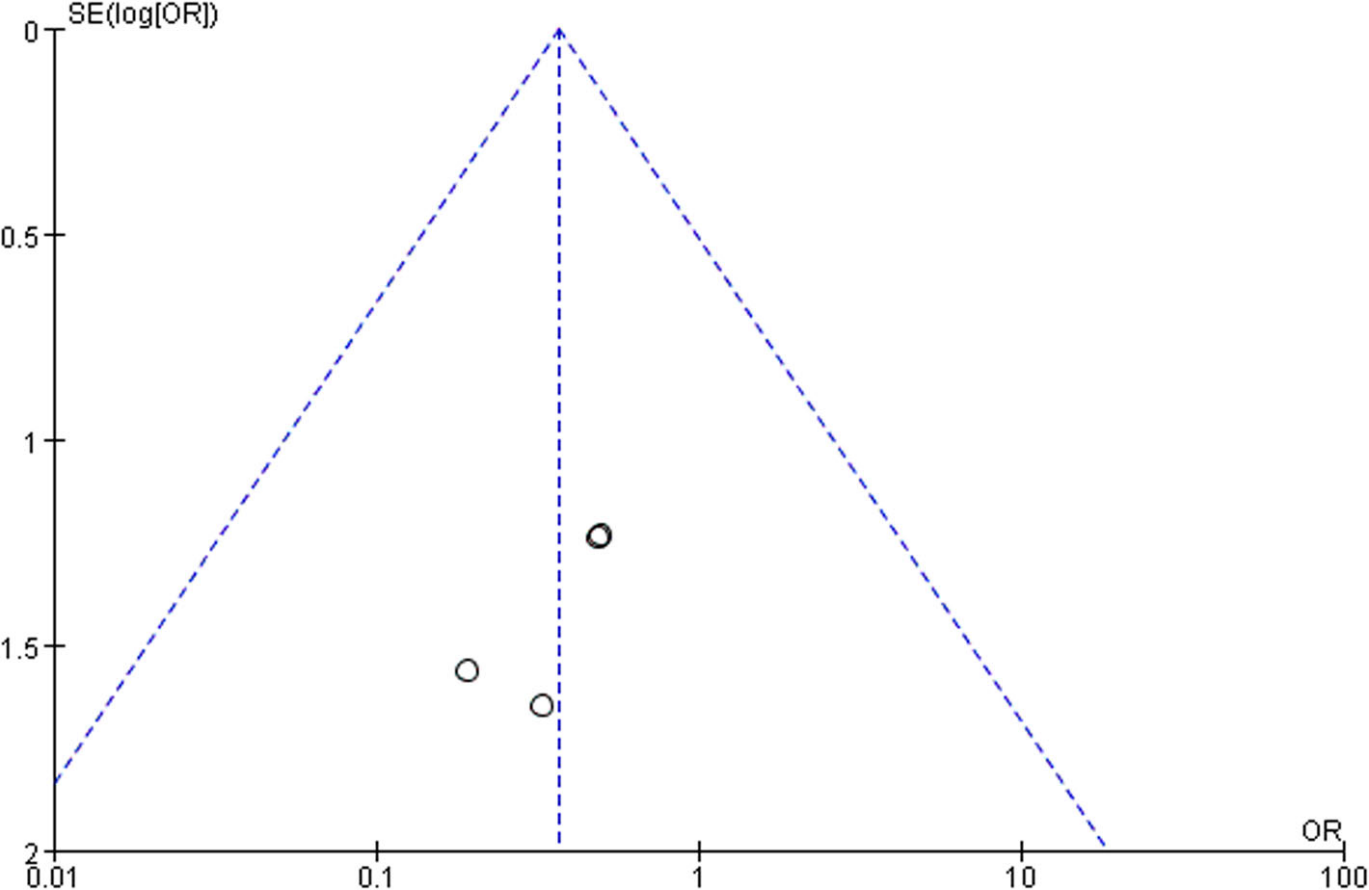
DVT funnel plot

**Fig. 13:**
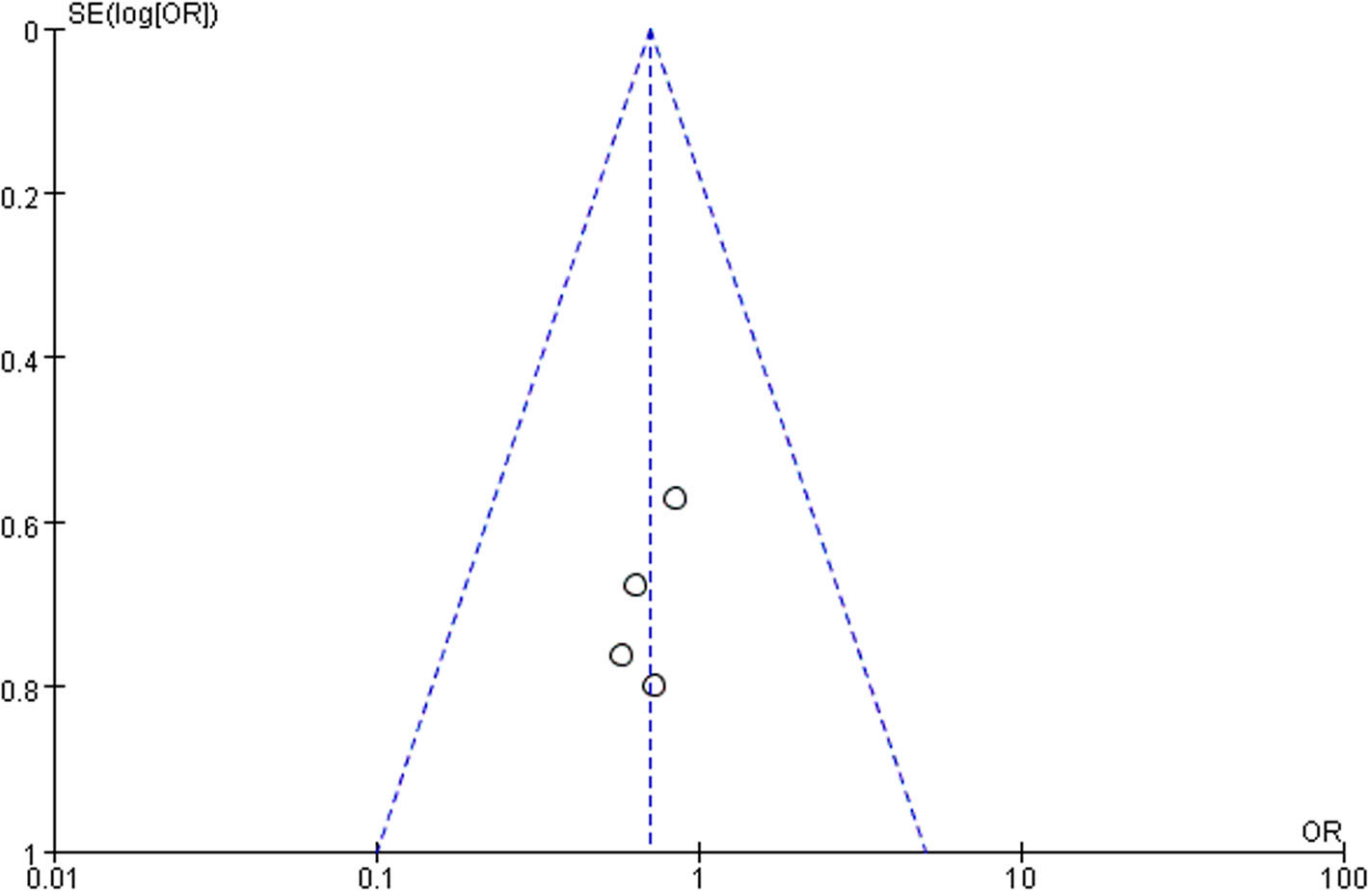
IVT funnel plot

**Fig. 14:**
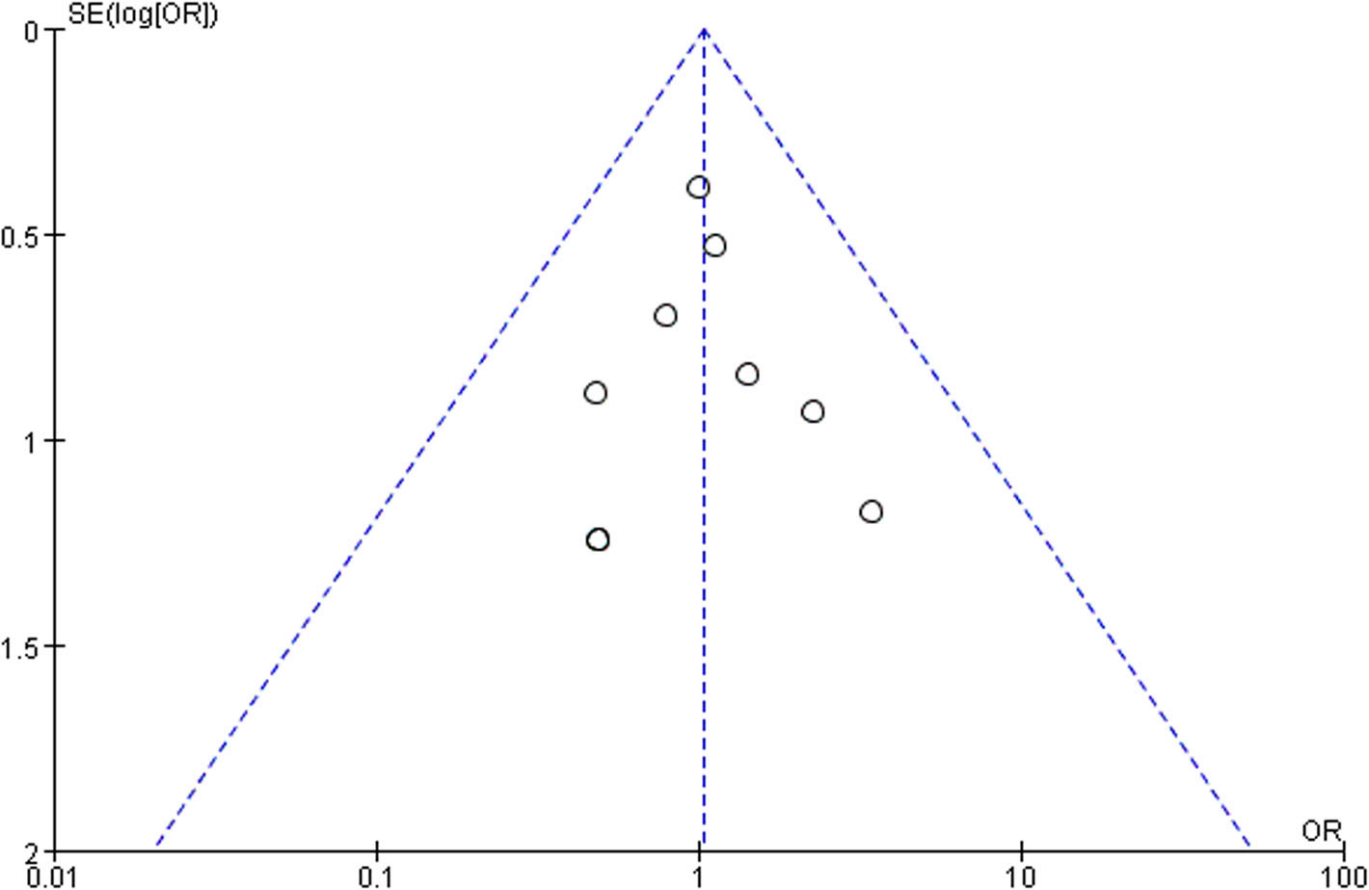
Transfusion rate funnel plot

Ten studies involving 1140 patients were included in this meta-analysis; compared with intravenous administration of TXA, oral TXA have similar outcomes in the total blood loss (TBL) [MD = − 3.67, 95% CI (− 45.12 to 37.78), *P* = 0.86], decline of hemoglobin (DHB) [MD = − 0.03, 95% CI (− 0.11 to 0.05), *P* = 0.45], length of hospital stay (HS) [MD = 0.09, 95% CI (− 0.10 to 0.27), *P* = 0.36] and there have no significant difference in terms of DVT [OR = 0.37, 95% CI (0.10 to 1.40), *P* = 0.14], IVT [OR = 0.71, 95% CI (0.36 to 1.38), *P* = 0.31], and transfusion rate [OR = 1.03, 95% CI (0.65 to 1.61), *P* = 0.91], but five studies suggested that oral TXA significantly reduced TXA cost compared with intravenous application (Figs. 3, 4, 5, 6, 7, 8, 9, 10, 11, 12, 13, and 14).

## Discussion

For TXA, our findings showed that compared with intravenous administration, oral application resulted in a lower TXA cost for total joint replacement patients. However, there were similar outcomes in terms of total blood loss (TBL), the decline in hemoglobin (DHB), and the length of hospital stay (HS) between the two treatment groups. In addition, the incidence of DVT and IVT and the transfusion rate between the two treatment groups showed no significant differences. The results suggested that oral TXA application is cheaper than intravenous administration and has the same efficacy and safety. Furthermore, oral TXA is a simpler procedure for reduced blood loss. Thus, we considered oral TXA to be more accepted for total joint replacement patients.

Blood loss is a common complication for total knee and hip arthroplasty patients that might result in swelling, stiffness, and delayed rehabilitation. Several techniques have been used for reduced blood loss during surgery, and allogeneic blood transfusions are a popular method for improving patients’ postoperative HB levels. Because they inhibit the immune system and increase the chance of infection, various risks of transfusion have been reported in previous studies. Thus, there is an urgent need for a more effective and safer method [25–28]. TXA is an anti-fibrinolytic drug that has been demonstrated to effectively reduce blood loss in TKA and THA patients in many publications. However, the majority of reports focused on topical and intravenous TXA administration. Previous studies have confirmed that there is similar effectiveness and safety in reducing total blood loss and transfusions between the two treatments, but little is known about the use of oral TXA in primary total joint replacement. Alipour et al. [29] reported that 1 g oral TXA applied 2 h preoperation significantly reduced blood loss compared with the placebo group. Lee et al. [30] demonstrated that patients who received oral TXA administration showed less total blood loss than placebo patients. Although Irwin et al. [31] reported that the oral TXA group (2.5 g/dL) had a greater change in decline in Hb than the IV TXA group (2.3 g/dL), the credibility of the results posed a challenge due to the retrospective design. Thus, to ensure the accuracy of the results, only RCTs were included in this research, and our results suggested that oral TXA was equally effective in terms of total blood loss and decline in HB compared with IV TXA. Additionally, five studies [15–17, 19, 21] have confirmed the cost-effective benefits of oral TXA (O: $68.11/68.11/85.14/274.81/77.48; IV: $472.44/474.1/447/860.28/648.96).

Transfusion is also a major problem; more blood loss during TKA or THA often results in transfusion, and Johansson et al. [32] found that intravenous TXA could significantly reduce blood loss and the transfusion cost during total hip arthroplasty compared with the placebo group. Husted et al. [33] performed RCT studies to evaluate the effectiveness of TXA for primary THA patients, and the results showed that intravenous TXA achieved less blood loss and a lower transfusion rate compared with the control group. Performing a retrospective analysis of 1018 patients, Gillette et al. [34] showed that TXA has economic benefits in terms of pharmacy, blood, laboratory, and room and board costs. In addition, previous studies have indicated that oral TXA is also an effective method for preventing blood loss, but there is a limited research focus on comparing the transfusion rate between oral and IV TXA administration. Our findings indicated that oral TXA is equally effective in reducing the transfusion rate compared with IV TXA for primary total joint replacement patients. Furthermore, the length of hospital stay could reflect the progress of patient postoperative recovery and the economic impact. Five studies reported the days of hospital stay, and our results showed that there were no statistically significant differences between the IV and oral TXA groups. Based on the above results, we believe oral TXA has similar safety and effective outcomes in total joint replacement patients compared with IV TXA administration.

To the best of our knowledge, there is still no consensus regarding the optimal timing and dosing of IV and oral TXA. In our research, the included studies have shown different standard administrations of IV and oral TXA. Five studies recommended only one dose of IV or oral TXA preoperation, while five other studies adopted multiple doses of TXA administered at different time points between pre- and postoperation. The repeated administrations included two, three, and four times. In addition, there was no standard dose in TXA application. The included studies showed that the total oral TXA dose was 1950 mg, 2 g, or 3 g and that the single dose was 10 mg/kg, 15 mg/kg, or 20 mg/kg in IV TXA groups. Because of the different TXA timing and dosing standards applied in the included studies, we believe that it is difficult to determine the best procedure based on current data, but through our gross analysis, the results demonstrated that a total of 2 g oral TXA was an effective way of reducing blood loss during total joint replacement regardless of whether single or multiple dose administration is carried out. Although some researchers considered that multiple- or high-dose TXA administration can prevent drug concentration and show better outcomes, there is a need for high-quality RCT studies in the future to support this view [35, 36].

Many high-quality RCTs have demonstrated safety and efficacy based on different routes of TXA administration compared to placebo groups during TKA or THA, but various complications were also concerns for surgeons, especially the incidence of deep vein thrombosis (DVT) and intramuscular venous thrombosis (IVT). In addition, intravenous TXA could be associated with cardiovascular disease or renal dysfunction [37]. However, our results demonstrated that there were no significant differences in terms of the incidence of DVT and IVT between the IV and oral TXA treatments. Thus, we believe that oral TXA did not increase the risk of DVT and IVT occurrence compared with IV administration.

However, this meta-analysis has some limitations. First, this meta-analysis included ten studies; the surgeons came from different countries, and the surgical procedures and experiences were different, which may have caused bias in the results. Second, the estimated total blood loss and transfusion depended on different rules, which may have affected the accuracy of the final results. Third, all included studies lacked long-term follow-up, which might have decreased the reliability of the results. Although this meta-analysis contains several limitations, all the included studies have an RCT design, which means that they are still powerful enough to guide future clinical work. Multicenter, prospective, randomized control trials with large sample sizes are needed in the future.

## Conclusion

In conclusion, our findings suggest that compared with the intravenous use of TXA, oral application has similar clinical outcomes in terms of total blood loss, the decline in hemoglobin, the length of hospital stay, the incidence of DVT and IVT, and the transfusion rate and a lower expense.

## Data Availability

All authors consent for the availability of data and materials.

## Abbreviations

CIs: Confidence intervals
DHB: Decline of hemoglobin
DVT: Deep vein thrombosis
HS: Hospital stay
IVT: Intramuscular venous thrombosis
RRs: Relative risks
TBL: Total blood loss
TKA/THA: Total knee/hip arthroplasty
TKR/THR: Total knee/hip replacement
TXA: Tranexamic acid
